# Exploring the utility of social-ecological and entomological risk factors for dengue infection as surveillance indicators in the dengue hyper-endemic city of Machala, Ecuador

**DOI:** 10.1371/journal.pntd.0009257

**Published:** 2021-03-19

**Authors:** Catherine A. Lippi, Anna M. Stewart-Ibarra, Timothy P. Endy, Mark Abbott, Cinthya Cueva, Froilán Heras, Mark Polhemus, Efraín Beltrán-Ayala, Sadie J. Ryan

**Affiliations:** 1 Quantitative Disease Ecology and Conservation (QDEC) Lab Group, Department of Geography, University of Florida, Gainesville, Florida, United States of America; 2 Emerging Pathogens Institute, University of Florida, Gainesville, Florida, United States of America; 3 Inter-American Institute for Global Change Research, Department of Montevideo, Montevideo, Uruguay; 4 Institute for Global Health and Translational Studies, State University of New York (SUNY) Upstate Medical University, Syracuse, New York, United States of America; 5 Department of Medicine, State University of New York (SUNY) Upstate Medical University, Syracuse, New York, United States of America; 6 Department of Microbiology and Immunology, State University of New York (SUNY) Upstate Medical University, Syracuse, New York; 7 Coalition for Epidemic Preparedness Innovations (CEPI), Washington, D.C., United States of America; 8 Universidad Técnica de Machala, Machala, Ecuador; Centers for Disease Control and Prevention, UNITED STATES

## Abstract

The management of mosquito-borne diseases is a challenge in southern coastal Ecuador, where dengue is hyper-endemic and co-circulates with other arboviral diseases. Prior work in the region has explored social-ecological factors, dengue case data, and entomological indices. In this study, we bring together entomological and epidemiological data to describe links between social-ecological factors associated with risk of dengue transmission at the household level in Machala, Ecuador. Households surveys were conducted from 2014–2017 to assess the presence of adult *Aedes aegypti* (collected via aspiration) and to enumerate housing conditions, demographics, and mosquito prevention behaviors. Household-level dengue infection status was determined by laboratory diagnostics in 2014–2015. Bivariate analyses and multivariate logistic regression models were used to identify social-ecological variables associated with household presence of female *Ae*. *aegypti* and household dengue infection status, respectively. *Aedes aegypti* presence was associated with interruptions in water service and weekly trash collection, and household air conditioning was protective against mosquito presence. Presence of female *Ae*. *aegypti* was not associated with household dengue infections. We identified shaded patios and head of household employment status as risk factors for household-level dengue infection, while window screening in good condition was identified as protective against dengue infection. These findings add to our understanding of the systems of mosquito-borne disease transmission in Machala, and in the larger region of southern Ecuador, aiding in the development of improved vector surveillance efforts, and targeted interventions.

## Author summary

The management of dengue fever, spread by the mosquito *Aedes aegypti*, is a major challenge to public health in southern coastal Ecuador. This is particularly true in the city of Machala, Ecuador, where the annual number of dengue fever cases is historically high. In this study, we bring together mosquito survey and dengue human infection data to describe household characteristics that are associated with dengue risk in Machala. Household surveys were conducted to determine the presence of adult *Ae*. *aegypti* mosquitoes, household-level dengue infection status, and to collect information on housing conditions, members of the household, and mosquito prevention behaviors. Statistical analyses were performed to determine which household characteristics were linked to presence of female *Ae*. *aegypti* mosquitoes and dengue infection status. We identified several factors that were associated with increased risk of household mosquito presence, or dengue infections, in addition to protective characteristics associated with lower risk. Interestingly, presence of female *Ae*. *aegypti* did not predict which households would have dengue infections. These findings add to our understanding of mosquito-borne disease risk in Machala, and in the larger region of southern Ecuador, aiding in the development of improved mosquito and arbovirus control and surveillance efforts.

## Introduction

The management of arthropod-borne viruses (arboviruses) is a fundamental task of public health agencies throughout much of Latin America and the tropics. Pathogens transmitted by the mosquito *Aedes aegypti*, such as dengue virus, impose some of the greatest growing disease burdens in this region [1]. Dengue has increased 30-fold over the past 50-years, globally and in South America. Over the past decade more than 19.6 million cases of dengue infection were reported in the Americas [2–4]. Dengue, like many arboviral infections, has limited options for clinical treatment and marketable vaccines, leaving vector control as a primary means of preventing and mitigating large outbreaks [5–7]. While there are many established protocols for designing and conducting public health vector control activities, these are subject to logistical constraints, resource availability, and fluctuating funding streams [6–11]. Development of targeted vector control strategies that focus on surveillance indicators, or known risk factors for transmission, offer a possible solution for implementing effective control measures in resource-limited communities (e.g., Lippi et al. 2020) [12]. Yet, social and ecological risk of exposure to arboviral infections is often understudied at fine spatial scales, such as individual households, at which many vector control interventions operate. Household-level features and behaviors can vary greatly within the built environment leading to varying risk of exposure to vectors, and thus, transmission [13–15].

Entomological and epidemiological surveillance systems can be integral components of public health vector control programs, where incoming data are key for informing management strategies and developing policy [11,16,17]. Vector abundance data are often used by public health agencies to trigger vector control responses, though the utility of directly inferring transmission risk from *Ae*. *aegypti* densities is difficult to establish, and varies widely across surveillance programs [17–19]. Beyond informing public agency responses, surveillance indicators can be used in the broader context of modeling risk by establishing correlative links between health outcomes and the social-ecological conditions in which they occur [20,21]. Ecological studies that establish such connections between dengue and factors such as population density, socioeconomic status, and housing conditions are routinely seen in the public health literature [14,20,22–24]. While observational studies such as these are inappropriate for inferring causality, they remain useful for informing current decision-making and guiding further epidemiological investigations.

**Fig 1:**
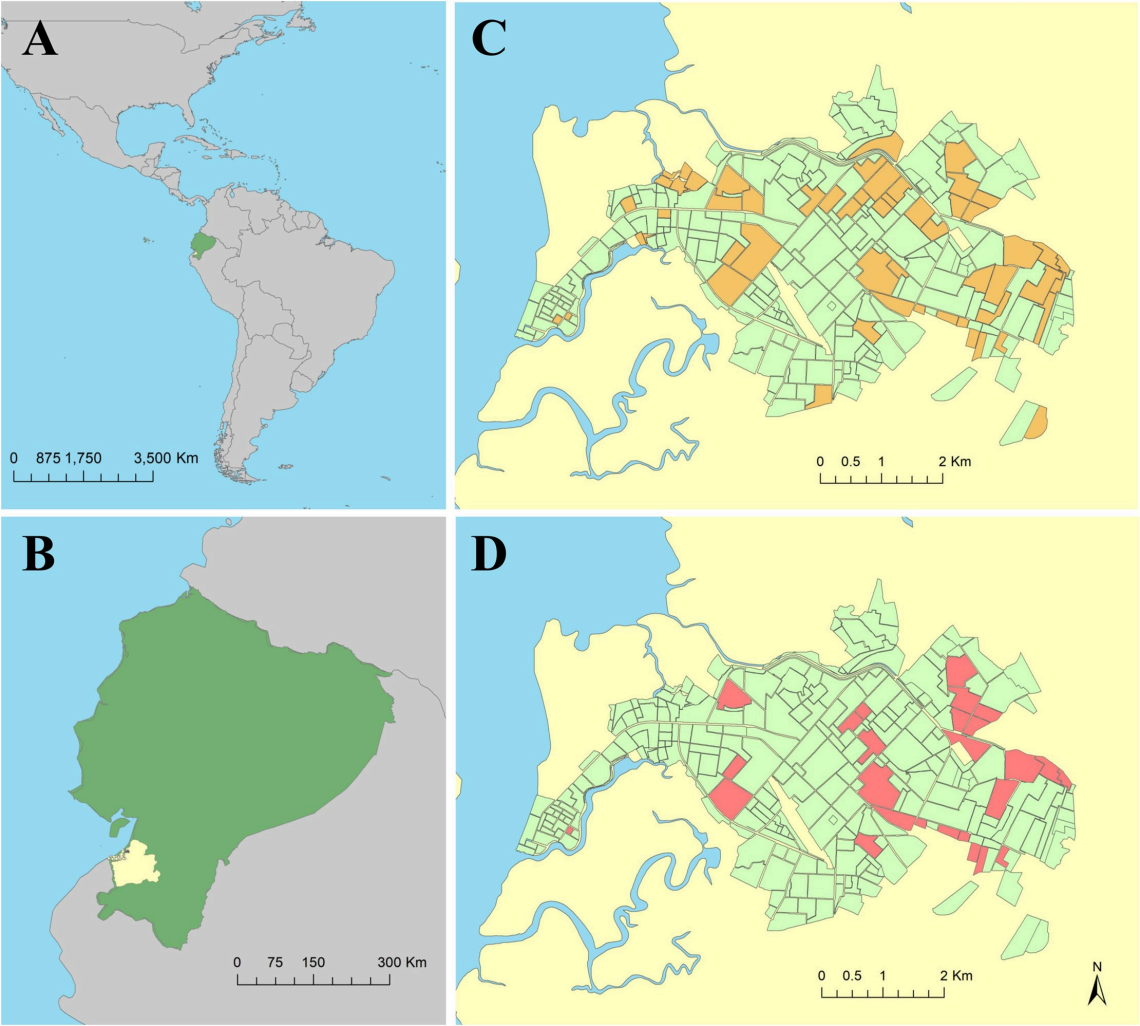
The study was conducted Machala, a city in the South American (A) country of Ecuador (B), located in El Oro province (B, shown in yellow). Households sampled during this study were located throughout the city of Machala, consisting of 460 households in 94 sampling clusters surveyed for Aedes aegypti mosquitoes (C), and 141 households in 33 sampling clusters surveyed for both Ae. aegypti mosquitoes and dengue infection status (D). Household locations were aggregated to census block for de-identification purposes in these figures. This figure was produced in ArcMap 10.6.1 (ESRI, Redlands, CA) using shapefiles freely available from the Natural Earth dataset ver. 4.1.0 (naturalearthdata.com) and georeferenced surveillance data provided by the MSP and edited by CAL.

In Ecuador, the national public health agency, the Ministerio de Salud Pública (MSP), oversees vector control, entomological surveillance, and epidemiological surveillance initiatives [25,26]. Ecuador’s southern coastal region is an area of historically high mosquito-borne disease activity [27,28]. El Oro province, located on the border with Peru, is consistently burdened with dengue fever outbreaks, where four serotypes of dengue virus are in circulation (DENV1-4), and more recently, chikungunya and Zika viruses [27]. The port city of Machala, with an estimated population of over 280,000, is Ecuador’s fourth largest city, the capital of El Oro province, and a major epicenter of commercial agricultural trade (Fig 1) [29]. Machala has notably high dengue fever incidence compared to other municipalities in Ecuador, where annual incidence rates reported to the MSP were 42.6 cases per 10,000 people in 2014 [27].

Dengue is hyperendemic in Machala, where *Ae*. *aegypti* is prevalent and transmission can occur throughout the year [30,31]. The high incidence of dengue, large population, and longstanding history of vector control and surveillance activities make Machala an ideal setting for studying arboviral transmission systems. Several previous studies conducted in Machala have assessed risk factors for dengue fever outbreaks at the mesoscale (e.g. neighborhoods or census blocks), including larval vector densities, environmental drivers, sociodemographic characteristics, community perceptions, and service accessibility [12,14,25,30,32–34]. The findings of these studies are useful in a public health advisory capacity, providing information that guides decision-making and research. Nevertheless, studies conducted on data aggregated to administrative units face limitations in terms of guiding spatially explicit interventions on fine scales, and we cannot assume that neighborhood-level associations will be observable at the level of households, where people are exposed to mosquitoes [35–37], and at which scale interventions such as fogging (household insecticidal spraying), or individual prevention behaviors occur.

The objective of this study is to identify relationships between household social-ecological factors and adult *Ae. aegypti* presence and dengue cases, respectively, in Machala, Ecuador. We also test if *Ae. aegypti* presence is an indicator of household dengue fever cases. Household characteristics associated with the dengue fever transmission cycle are potentially identifiable targets for public health vector control intervention to reduce mosquitoes.

## Methods

### Cluster study design

The data used in this study were collected as part of a SUNY Upstate Medical University epidemiological study of dengue in Machala, Ecuador conducted from 2014–2017. The study protocol for collection of human case data was reviewed and approved by the Institutional Review Boards at the State University of New York (SUNY) Upstate Medical University, Cornell University, the Human Research Protection Office of the U.S. Department of Defense, the Luis Vernaza Hospital in Guayaquil, Ecuador, and the Ecuadorian MSP. Four sentinel clinics operated by the MSP were selected to enroll households into the study. These clinics were chosen based on historical patterns of dengue detection and available medical resources. A maximum of four index cases were randomly selected each week from clinically diagnosed dengue fever cases passively detected in sentinel healthcare facilities. A cluster study design was used to enroll additional participants. After identifying the households of index cases, up to four additional households were enrolled within a 200m radius of each index house, representing the approximate flight range of *Ae*. *aegypti* mosquitoes; this study protocol has been described in detail previously [38]. Each household was visited once during the study, within two days of enrollment, at which time household survey data, entomological data, and blood samples from individuals were collected. Locations of enrolled households (i.e. latitude and longitude coordinates) were recorded on site using Garmin handheld global positioning system (GPS) units.

*Household survey*–Field teams comprising local Ecuadorians visited each enrolled household, administering a survey to the head of household. Survey questions were developed based on previous studies from the region, which identified potential local factors of interest as they relate to risk of dengue transmission [30]. The survey tool used in this study can be found in the supporting information from Kenneson et. al [39,40]. The survey was used to collect information on household demographics, socioeconomic status, access to municipal services, and human behaviors that may influence the presence of mosquitoes (e.g. water storage practices and use of pesticides). Field teams also visually assessed housing conditions and structures, collecting data on patio conditions, window screening, ventilation method (i.e. fan or air conditioning unit), and construction materials. In addition to presence, the quality of certain structures was categorized by technicians on site, where features were considered to be in “poor” condition if they could not exclude mosquitoes from the household (e.g. window screens with visible holes) or provided ovipositional sites for *Ae*. *aegypti* mosquitoes (e.g. patios with collected garbage). Patios were additionally categorized as “shaded” if 50% of more of the area was covered by large tree canopies or greenhouse cloth. General housing condition was categorically ranked by technicians on site, ranging from “good” housing, which consisted of new construction in good repair (e.g. no holes in walls, well-maintained roof, windows and doors that shut, etc.), to “poor” conditions where structures were not maintained (e.g. older buildings in disrepair, observable damage to structure, holes in walls, unpainted exteriors, etc.). The complete list of household variables used in this study, with definitions, is provided in S1 Table.

### Entomological survey

Field teams of entomology technicians visited households enrolled in the cluster study from 2014–2017. Households were surveyed for mosquitoes within two days of enrollment to assess abundance and presence of *Ae*. *aegypti*. Sampling consisted of backpack aspirator (Prokopack) collection of adult mosquitoes in and around households [41]. The entomological sampling protocol consisted of one technician operating the backpack aspirator and sampling the intradomicile (i.e. within the home) for 20 minutes and the peridomicile (i.e. courtyard or patio associated with home) for 10 minutes. Each room within a given household was sampled, starting at the floor, sampling under furniture, and working up to the ceiling. Adult mosquitoes were stored in coolers in the field, then identified, counted, and sorted by sex and species (i.e. *Ae*. *aegypti* versus other) at the entomological lab at the Technical University of Machala. Abundance counts of female *Ae*. *aegypti* for each year of the study were aggregated by households into a binary (i.e. presence/absence) dataset for statistical modeling (S1 Data).

### Human case data

Data on dengue infection status were collected for enrolled households from 2014–2015; diagnostic results were not available for 2016–2017. Prior to initiating the study, all participants, or adult legal guardians, engaged in a written informed consent process. Cases detected via passive clinical surveillance were informed that they may be randomly selected to participate in the cluster study prior to signing the informed consent. The study included children (> 6 months) to adults who were evaluated for DENV infection.

Laboratory diagnostic tests for DENV were run on blood samples collected from study participants. These included NS1 rapid strip tests (Panbio Dengue Early Rapid), ELISA assays for NS1 (Panbio Dengue Early ELISA), IgM immunoassays (Panbio Dengue Capture IgM), and RT-PCR. Full details of the diagnostic testing used in this study are outlined in Stewart-Ibarra et al. (2018) [27]. Household members were considered positive for DENV infection if at least two diagnostic tests were positive. Case counts for each given year of the study were aggregated by households into a binary dataset for statistical modeling.

### Statistical analysis

Statistical analyses and modeling were performed in R (ver. 3.6.2). Intraclass correlation coefficient values were calculated for the outcomes of *Ae*. *aegypti* presence and DENV infections, and indicated that household clusters did not impact observed variance in the data [42]. Bivariate analysis of social-ecological survey variables was conducted via Fisher’s exact test, testing for significant factors influencing the household presence of female *Ae*. *aegypti* mosquitoes or dengue cases, respectively. Statistically significant variables were used as predictors in a generalized linear model (GLM) framework, specifying a logistic modeling distribution (GLM, family = binomial, link = logit), to assess combined household-level influences on outcomes of interest (i.e. mosquito presence or presence of dengue cases).

Variance inflation factors (VIF) were calculated to evaluate multi-collinearity in household survey variables, with values below 10 indicating low collinearity [43]. Stability of logistic regression models was assessed via condition numbers (k), where values below 30 indicate stable models [44]. Global Moran’s I with inverse distance weighting was performed in ArcMap (ver. 10.6.1, ESRI, Redlands, CA, USA) on model residuals to test for presence of spatial autocorrelation, a violation of GLM assumptions [45].

## Results

**Fig 2:**
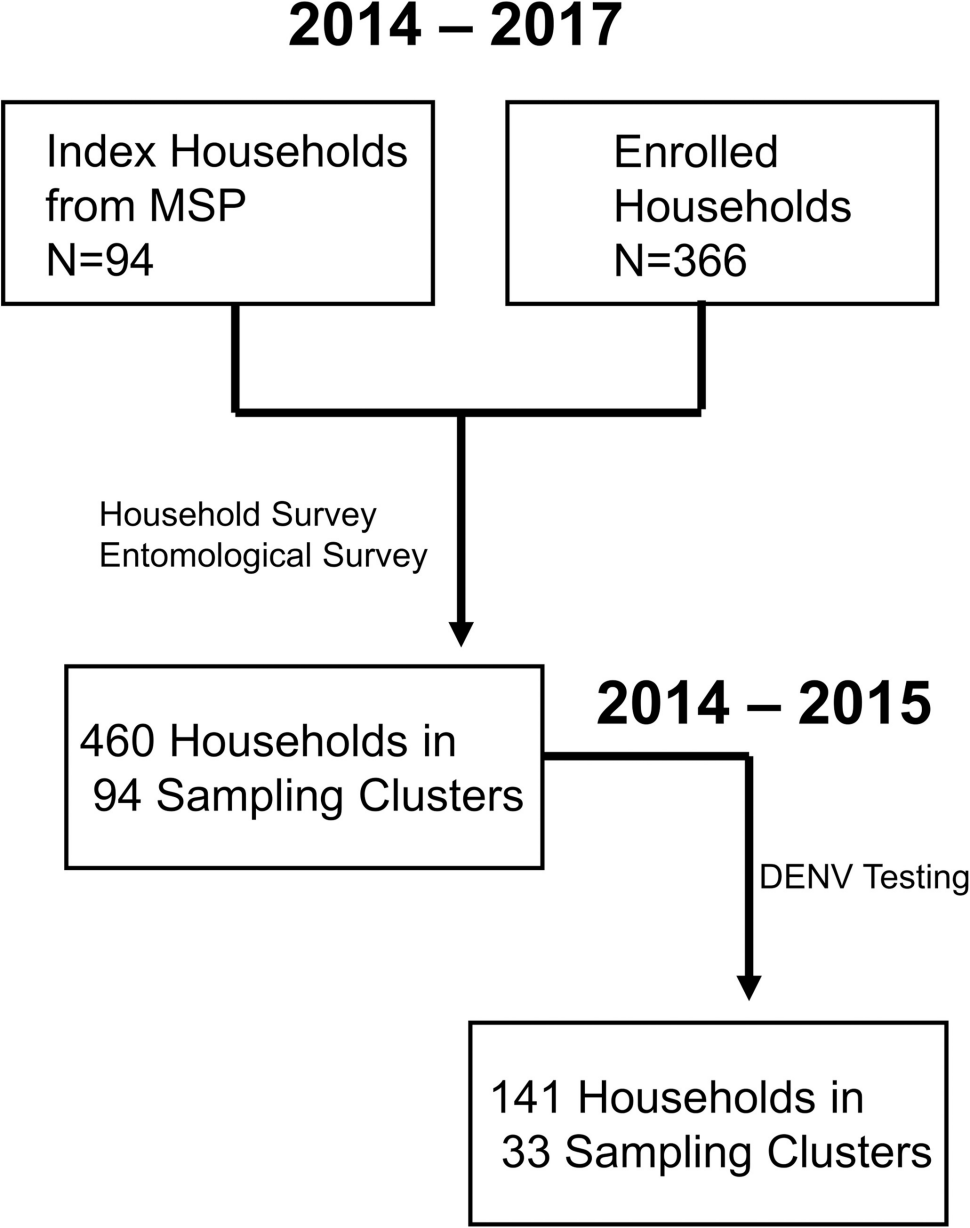
Diagram of household enrollment and data collection for cluster study design in Machala, Ecuador.

Ninety-four cases with lab-confirmed acute symptomatic DENV infections were randomly selected for this study via passive surveillance at MSP sentinel facilities during the 2014–2017 study period. These index cases were used to subsequently enroll an additional 366 households into the cluster study, for a total of 460 households in Machala, spanning 94 sampling clusters surveyed for household risk factors and mosquito presence during the study (Table 1, Figs 1C and 2). *Aedes aegypti* mosquitoes were collected in 54.1% of households (n = 249), and 43.9% of households (n = 202) had female *Ae*. *aegypti* present.

**Table 1:** Summary of social-ecological variables collected from 2014–2017 household surveys in Machala, Ecuador. Dengue survey households represent a subset of households with valid diagnostic test results and complete household and entomological survey data.

Parameter	Households (n = 460)	%	Households (n = 141)	%
	**Entomological Survey**		**Dengue Survey**	
**Housing conditions **				
Good Condition Housing	144	32.0%	55	39.0%
Poor Condition Housing	55	12.0%	16	11.3%
Cane House Construction	32	7.2%	9	6.4%
Wood House Construction	9	2.0%	2	1.4%
House is Rented	40	9.5%	15	10.6%
Piped Water in Household	320	76.0%	100	70.9%
Interruptions in Piped Water	245	53.3%	78	55.3%
Municipal Garbage Collection	400	95%	130	92.2%
Municipal Sewage	356	84.8%	119	84.4%
Septic Tank	54	12.8%	20	14.2%
Air Conditioning	30	7.1%	13	9.2%
Uses a Fan	164	39.0%	70	49.6%
Screens on Windows	102	22.8%	44	31.2%
Window Screen in Good Condition	88	19.7%	35	24.8%
Access to Paved Roads	278	62.2%	68	48.2%
Standing Water Present	213	49.5%	72	51.1%
Adjacent to Abandoned Housing	119	26.6%	49	34.8%
Patio Present	367	82.1%	111	78.7%
Patio in Bad Condition	81	18.1%	21	14.9%
Patio Shaded (> 50%)	56	12.5%	19	13.5%
**Household Demographics and Practices**				
Head of Household (HOH) Employed	359	85.7%	120	85.1%
HOH male	323	76.7%	103	73.0%
HOH Earns Less than Minimum Wage	74	17.6%	11	7.8%
HOH Has Secondary Education	161	38.2%	57	40.4%
Stores Water	283	67.2%	96	68.1%
Know mosquitoes transmit dengue	372	88.8%	124	87.9%
Know standing water produces mosquitoes	402	95.9%	136	95.7%
Uses Larvicide	98	23.3%	47	33.3%
Uses Abate	151	35.9%	2	1.4%

Housing conditions vary considerably in Machala, with approximately one third (32%) of surveyed housing classified in good condition, and 12% of housing structures classified in poor condition. Access to municipal services among sampled households was high, with 62.2% accessible by paved roads, 76.0% having access to piped water, 84.8% with access to sewage, and 95.0% receiving garbage collection services. The head of household was employed in 85.3% of surveyed households, and 17.6% of heads of household earned less than the minimum wage.

Four hundred study participants from enrolled households were tested for dengue infection status during 2014–2015, and 311 of these had valid diagnostic test results and complete household and entomological survey data. Infection status during 2014–2015 was aggregated to the household level for 141 households in 33 sampling clusters (Table 1, Fig 1D). Of these households, 29.08% had one or more household members positive for dengue. All four DENV serotypes were detected in Machala during the study period, and of the thirty-two households tested for dengue serotype, DENV-2 (56.3%) and DENV-1 (40.6%) were the most prevalent infections. *Aedes aegypti* mosquitoes were collected in 57.4% of the subset of households (n = 81) that were tested for dengue infection, and 49.6% of these households had female *Ae*. *aegypti* present (n = 70).

Housing conditions, demographics, and practices were statistically compared for households where female *Ae*. *aegypti* were found, or were not found, in the entomological survey (Table 2). Significant (p<0.05) factors positively associated with mosquito presence were interruptions in piped water availability, municipal garbage collection, access to paved roads, presence of standing water, and having a patio in poor condition. Home air conditioning, window screening, window screening in good condition, and adjacency to abandoned housing were negatively associated with female *Ae*. *aegypti* presence.

**Table 2:** Social-ecological factors in households with versus without female *Aedes aegypti* present.

Parameter	Households (n = 202)	%	Households (n = 258)	%	p-value
	***Ae*. *aegypti* Present**		***Ae*. *aegypti* Absent**		
**Housing conditions **					
Good Condition Housing	53	26.24%	91	35.27%	0.324
Poor Condition Housing	28	13.86%	27	10.47%	0.313
Cane House Construction	9	4.46%	23	8.91%	0.065
Wood House Construction	3	1.49%	6	2.33%	0.737
House is Rented	20	9.90%	20	7.75%	0.617
Piped Water in Household	147	72.77%	173	67.05%	0.731
Interruptions in Piped Water	125	61.88%	120	46.51%	**0.001**
Municipal Garbage Collection	187	92.57%	213	82.56%	**0.013**
Municipal Sewage	166	82.18%	190	73.64%	0.278
Septic Tank	20	9.90%	34	13.18%	0.241
Air Conditioning	5	2.48%	25	9.69%	**0.001**
Uses a Fan	84	41.58%	80	31.01%	0.057
Screens on Windows	34	16.83%	68	26.36%	**0.013**
Window Screen in Good Condition	28	13.86%	60	23.26%	**0.009**
Access to Paved Roads	137	67.82%	141	54.65%	**0.008**
Standing Water Present	108	53.47%	105	40.70%	**0.010**
Adjacent to Abandoned Housing	42	20.79%	77	29.84%	**0.024**
Patio Present	167	82.67%	200	77.52%	0.321
Patio in Bad Condition	44	21.78%	37	14.34%	**0.049**
Patio Shaded (> 50%)	26	12.87%	30	11.63%	0.775
**Household Demographics and Practices**					
Head of Household (HOH) Employed	161	79.70%	198	76.74%	0.679
HOH male	145	71.78%	178	68.99%	0.730
HOH Earns Less than Minimum Wage	35	17.33%	39	15.12%	0.797
HOH Has Secondary Education	75	37.13%	86	33.33%	0.763
Stores Water	125	61.88%	158	61.24%	0.532
Know mosquitoes transmit dengue	168	83.17%	204	79.07%	0.645
Know standing water produces mosquitoes	180	89.11%	222	86.05%	0.346
Uses Larvicide	42	20.79%	56	21.71%	0.643
Uses Abate	72	35.64%	79	30.62%	0.477

P-values in bold denote statistical significance found via Fisher’s exact test

Statistically significant variables from bivariate analyses were put into a multivariate logistic regression model for female *Ae*. *aegypti* presence (Table 3). Water interruptions (OR = 1.67) and garbage collection (OR = 3.29) remained significant risk factors for household *Ae*. *aegypti*, while air conditioning (OR = 0.28) was associated with reduced presence. Other variables were no longer significant in the multivariate model. Low condition number indicated model stability (k = 7.15).

**Table 3:** Logistic regression model of household female *Aedes aegypti* presence in Machala, Ecuador.

Model	Estimate	SE	P-Value	OR	95% CI
Intercept	-1.81	0.63	0.004	-	-
Water Interruptions	**0.51**	**0.21**	**0.017**	**1.67**	**1.10–2.55**
Trash Collection	**1.19**	**0.59**	**0.043**	**3.29**	**1.13–11.98**
Air Conditioning	**-1.27**	**0.52**	**0.014**	**0.28**	**0.09–0.72**
Screens Present	-0.13	0.34	0.697	0.88	0.45–1.71
Screens in Good Condition	-0.40	0.36	0.263	0.67	0.33–1.35
Paved Roads	0.45	0.23	0.053	1.56	0.10–2.46
Standing Water Present	0.25	0.23	0.264	1.29	0.83–2.01
Adjacent to Abandoned Property	-0.37	0.25	0.141	0.69	0.42–1.13
Patio in Poor Condition	0.23	0.29	0.426	1.26	0.72–2.22

k = 7.15, pseudo R^2^ = 0.09

Housing conditions, demographics, and practices were statistically compared for households with DENV infections versus households without DENV infections (Table 4). Significant (p<0.05) factors positively associated with household DENV infections were having a shaded patio and HOH employment. Households with window screening in good condition were negatively associated with presence of DENV infections. Female *Ae*. *aegypti* presence in households was not significantly associated with presence of DENV infections.

**Table 4:** Social-ecological factors in households with versus without dengue infections.

Parameter	Households (n = 41)	%	Households (n = 100)	%	P-value
	**Dengue Positive**		**Dengue Negative**		
**Housing conditions **					
Good Condition Housing	11	26.83%	44	44.0%	0.061
Poor Condition Housing	7	17.07%	9	9.0%	0.240
Cane House Construction	5	12.20%	4	4.0%	0.122
Wood House Construction	1	2.44%	1	1.0%	0.499
House is Rented	6	14.63%	9	9.0%	0.371
Piped Water in Household	26	63.41%	74	74.0%	0.225
Interruptions in Piped Water	19	46.34%	59	59.0%	0.194
Municipal Garbage Collection	37	90.24%	93	93.0%	0.730
Municipal Sewage	32	78.05%	87	87.0%	0.206
Septic Tank	8	19.51%	12	12.0%	0.290
Air Conditioning	6	14.63%	7	7.0%	0.120
Uses a Fan	18	43.90%	52	52.0%	0.459
Screens on Windows	8	19.51%	36	36.0%	0.072
Window Screen in Good Condition	5	23.81%	30	30.0%	**0.032**
Access to Paved Roads	17	41.46%	51	51.0%	0.355
Standing Water Present	23	56.10%	49	49.0%	0.464
Adjacent to Abandoned Housing	18	43.90%	31	31.0%	0.174
Patio Present	33	80.49%	78	78.0%	0.824
Patio in Bad Condition	8	19.51%	13	13.0%	0.434
Patio Shaded (> 50%)	10	24.39%	9	9.0%	**0.027**
**Household Demographics and Practices**					
Head of Household (HOH) Employed	39	95.12%	81	81.0%	**0.037**
HOH male	31	75.61%	72	72.0%	0.835
HOH Earns Less than Minimum Wage	2	4.88%	9	9.0%	0.510
HOH Has Secondary Education	17	41.46%	40	40.0%	1.000
Stores Water	27	65.85%	69	69.0%	0.843
Know mosquitoes transmit dengue	38	92.68%	86	86.0%	0.395
Know standing water produces mosquitoes	40	97.56%	96	96.0%	1.000
Uses Larvicide	14	34.15%	33	33.0%	1.000
Uses Abate	1	2.44%	1	1.0%	0.499
**Entomological Survey**					
Female *Aedes aegypti* present	22	53.66%	48	48.0%	0.582

P-values in bold denote statistical significance found via Fisher’s exact test

Statistically significant variables from bivariate analyses were put into a multivariate logistic regression model for presence of household DENV infection (Table 5). Head of household employment (OR = 4.96) and households with shaded patios (OR = 3.81) were significant risk factors for household DENV infection. Window screening in good condition (OR = 0.30) was protective against presence of DENV infections. Low condition number indicated model stability (k = 4.75).

**Table 5:** Logistic regression model of household presence of dengue cases in Machala, Ecuador.

Model	Estimate	SE	P-Value	OR	95% CI
Intercept	-2.30	0.78	0.003	-	-
Screens in Good Condition	-1.20	0.55	0.028	0.30	0.09–0.82
Patio Shaded	1.34	0.54	0.014	3.81	1.33–11.47
HOH Employed	1.60	0.79	0.043	4.96	1.28–33.30

k = 4.75, pseudo R^2^ = 0.11

Intraclass correlation (ICC) values for both entomological and human case datasets were extremely low (i.e. near or equal to zero), indicating that the clustered study design did not meaningfully contribute to variance in the data. Despite the clustered arrangement of households in the study design, global clustering was not detected in model residuals for mosquito presence (Moran’s I = 0.04, p-value = 0.07) or dengue presence (Moran’s I = -0.14, p-value = 0.15), indicating no impact of spatial autocorrelation on model results.

## Discussion

This study provides a household-level assessment of dengue fever risk in Machala, Ecuador, a city with historically high burden of the disease. Indicators of mosquito presence are at times used by public health agencies as a proxy for risk of disease exposure and basis for vector control operations, particularly in absence of active human surveillance data [2,6]. This is an intuitive approach, as widespread surveillance of human cases is resource intensive, and vector presence is a necessary component of transmission cycles for pathogens like dengue virus [46,47]. Yet, despite the close association of *Ae*. *aegypti* with built urban and human environments, we could not find a distinct link between adult mosquito presence and dengue risk in Machala. Female *Ae*. *aegypti* presence was not identified as a significant household risk factor for dengue infection status. Interestingly, this finding parallels a previous study conducted by Stewart-Ibarra et. al during the 2010 dengue outbreak in Machala, where neighborhood larval mosquito indices were not predictive of dengue cases in the study’s best-fit model [30].

The lack of association between mosquito presence and dengue infections indicated in this study may reflect a disconnect between household conditions and locations where transmission is occurring, particularly in the context of hyperendemic dengue transmission in Machala. Studies conducted in other Latin American cities with hyperendemic dengue transmission have shown that within cities, transmission risk is influenced by neighboring census tracts, and no specific locations within a city were driving dengue cases [48,49]. Daily human movements and social connections within hyperendemic spaces can also drive dengue transmission, particularly on fine spatial scales [50,51]. Given the high prevalence of the disease in the community, risk of transmission in Machala may be more dependent on social and behavioral factors that increase exposure to infective mosquito bites, rather than mere presence or absence of the vector in the immediate vicinity of the household. Although not informative for dengue risk at the household level in this study, municipal and sub-regional entomological surveillance is still a cornerstone of successful public health vectors control programs, where mosquito counts are necessary to set goals and proactively trigger the deployment of vector control services ahead of epidemic peaks [6,52,53]. However, in locations with hyperendemic arboviral transmission, like Machala, surveillance of human cases may provide better primary information for guiding policy and developing direct community interventions on fine spatial scales.

We did not use mosquito abundance as a metric in modeling in this study due to low counts of adult mosquitoes (e.g. <10) recorded in household surveys via aspiration. Backpack aspiration is a logistically feasible surveillance method for many vector control agencies in Latin America. This method of sampling adult mosquitoes is relatively accessible, and there exists evidence of high sensitivity for detecting the presence of mosquitoes in households [18,41,54]. Regardless, indoor household *Ae*. *aegypti* densities can be notoriously low, and samples collected with backpack aspirators are often highly dependent on factors such as user skill and complexity of the indoor environment [55–57]. Some studies suggest that alternative trapping methods, such as the use of passive BG-Sentinel traps, may be better suited for targeting *Ae*. *aegypti* [9,56,57]. In contrast with binary presence data used here, adult mosquito abundance data have been shown in some instances to correlate with risk of dengue infection, particularly in longitudinal studies that can account for temporal variability and lagged effects [18,58].

Presence of *Ae*. *aegypti* was not predictive of dengue infections in Machala, yet mosquitoes are a necessary component of the dengue transmission cycle. Identification of factors that promote mosquito presence can provide valuable information for establishing mosquito control targets, validating or refining current MSP advisories and educational initiatives for the control of container-breeding mosquitoes [2,26]. We identified several factors related to water availability and housing conditions in our bivariate analyses that were associated with vector presence. These included interruptions in water service, presence of standing water around the household, patios in poor condition, municipal garbage collection, and paved road access. Air conditioning, proximity to abandoned housing, window screening, and window screening in good condition were negatively associated with mosquito presence. We did not find associations between mosquito presence and household demographics and practices, suggesting that measured social risk factors and personal protective behaviors for dengue did not reliably indicate local presence of adult female *Ae*. *aegypti* in Machala. Two risk factors, water interruptions and garbage collection, and one protective factor, household air conditioning, remained significant in the logistic regression model of female *Ae*. *aegypti* presence.

Associations between household characteristics and mosquito presence may be leveraged into actionable abatement strategies by the local public health agency, improving vector control and outreach efforts in Machala. Further, the overlap of risk factors with other studies may indicate candidate targets for regionally generalized mosquito control recommendations. For example, water availability and storage practices have previously been associated with immature mosquito presence in different cities, and at different spatial scales, in coastal Ecuador [14,59]. Protective measures may also be regionally transferrable. Factors that eliminate the need to open doors and windows for home ventilation, such as air conditioning, are typically found to be protective against exposure to mosquitoes across many systems [60–62]. Counterintuitive relationships observed at local scales, such as the positive association between weekly garbage collections and *Ae*. *aegypti* presence in Machala, are also informative to local mosquito control. Frequent municipal trash collection is generally associated with reduced debris, which translates to reduced ovipositional sites for container-breeding mosquitoes [63]. However, a census tract-level correlation between garbage collection and dengue fever was also observed in Guayaquil, Ecuador, suggesting that highly developed urban centers may provide ample mosquito production sites, and elevated disease risk, regardless of access to municipal services [20].

Household-level variables also influenced dengue infection status of homes in Machala. Window and house screening that was in good condition was identified as a significant protective factor against the presence of household dengue infections. Screened entryways can reduce exposure to potentially infective mosquitoes by establishing barriers to access of vectors to households [64]. The use of physical barriers to exclude insects is a cornerstone of integrated pest management, and has been effectively used to reduce dengue fever risk in many locations [64–67]. Yet, in Machala we found that presence of screening itself was not significantly protective against dengue. Screening in disrepair, where survey technicians reported visible damage or holes in screens, may not sufficiently exclude mosquitoes from households. We also identified the presence of shaded patios as a dengue risk factor in our study, a feature which has been linked to transmission risk in other studies in the region, promoting favorable habitat and oviposition sites for container-breeding mosquitoes [14,39].

The lack of association between household dengue cases and indicators of water availability found in this study is notable, and was unexpected. Frequent water interruptions promote water storage behaviors, such as collecting water in uncovered drums and basins, which increase suitable oviposition sites that can be exploited by *Ae*. *aegypti*. Mosquito reproduction and abundance are thus impacted by water storage practices, and interruption in water service was a significant predictor in the model for mosquito presence in this study. Access to piped water and water storage have been linked to both dengue infections and the proliferation of *Ae*. *aegypti* across multiple spatial scales, in Machala and other coastal Ecuadorian cities [14,20,30,31,68,69].

Head of household employment was a risk factor for dengue presence in this study. This relationship is counterintuitive, as HoH employment is often used as a proxy for higher socioeconomic status, which is generally associated with improved housing quality and lower arboviral disease risk [6,70]. However, occupational exposures to mosquito-borne diseases, where infective bites occur at the workplace, are known to vary across employment fields [71,72]. Although the survey tool used in this study represents a major step forward in the collection of fine-scale data regarding risk of dengue transmission in Ecuador, the questions did not necessarily capture the nuance of some risk elements. Events occurring beyond the home, tied to employment, such as those associated with occupation type, daily commuting patterns, or other work-related travel, were not captured by the survey tool. Such an association was reported in the Galapagos, where travel patterns were associated with self-reported dengue illness [45].

A previous study, conducted by Kenneson et al. (2017), also examined household dengue fever risk in Machala during 2014–2015 [39]. In the current study, we restricted analyses to households with complementary dengue case and entomological survey data, and used a more conservative and proximal dengue case definition, requiring at least two positive diagnostic tests, excluding IgG immunoassays, indicative of longer term exposure [4]. The 2017 study, with broader case definitions and a larger group of households, also found that shaded patios were significant risk factors for dengue fever, but additionally identified adjacency to abandoned properties and frequent water interruptions as significant risk factors. We found that frequent water interruption was a risk factor in our multivariate model for *Ae*. *aegypti* presence, but was not significant in the model for dengue case presence. While Kenneson et al. did not examine overall housing conditions, making direct comparisons unfeasible, they instead demonstrated that preventive actions and perceptions were negatively associated with dengue fever outcomes. While these studies leverage overlapping sampling events, the differences in objectives, case definitions, and drivers explored, indicate a need for further examination of the implications for connections in the system between the transmission conditions, the scale of human behavior, and household impact, and which components of the lived environment interact with the vector-human transmission cycle.

Household-level data are useful in advancing our understanding of localized disease transmission and exposure risk, yet there are caveats that must be considered before findings are used in an applied capacity. The statistical models used in this study are correlative, and we therefore must avoid assigning causality to any significant relationships without further investigation. Furthermore, data collected via household surveys certainly provide information on a fine scale, but they do not capture the full social-ecological transmission environment. This is exemplified by the lack of information concerning potential workplace and school exposures. We also caution against reducing localized risk indicators into a dichotomy of “poor” versus “affluent” neighborhoods; this fails to account for nuances in risk due to personal behaviors, or exposure due to hyperendemic presence of mosquito vectors. Focusing solely on economic indicators of risk has the additional potential to generate stigma in communities, which can undermine public health initiatives [73].

The results of this study aid us in identifying areas for further study as potential targets for developing proactive mosquito control activities and intervention strategies in Machala. The indication of missing links between entomological exposure risk indicators at the household level, where vector control intervention is often carried out, and the indicators of dengue infection, point to a need for a more complete examination of the social-ecological transmission environment. Nonetheless, comparing our findings to previously published work, we have identified commonalities in risk factors across spatial scales. Thus, we indicate that some household-level risk factors, such as water interruptions, may be generalizable in a policy development capacity.

## Conclusions

In this study, we sought to assess the utility of entomological surveillance, specifically backpack aspiration of adult *Ae*. *aegypti* mosquitoes, in the context of simultaneous epidemiological information about dengue, at the household level, in Machala, Ecuador. The use of backpack aspirators is an attractive option for entomological surveillance due to their relative accessibility. Yet, this method of surveillance faces a myriad of challenges including training technicians and gaining access to homes. Here, we found a mismatch between the entomological surveillance information for adult mosquitoes, the predictive capacity of those results in the context of risk, and identifiable social-ecological risk factors for dengue transmission. In situations where adult mosquito surveillance data are collected as an indicator of transmission risk, the utility of these data for identifying potential intervention targets should be evaluated. In Machala, where dengue is hyper-endemic, adult mosquito surveillance may not lead to major improvements in dengue risk assessment, particularly when compared to larval mosquito surveys and epidemiological surveillance conducted at broader scales.

## Supporting information

S1 Table Description of social-ecological variables collected in Machala via household surveys. (DOCX)

S1 Data Female *Aedes aegypti* presence detected in household entomological surveys conducted in Machala, Ecuador from 2014–2017. (XLSX)

## References

[1] Shepard DS, Coudeville L, Halasa YA, Zambrano B, Dayan GH. Economic impact of dengue illness in the Americas. Am J Trop Med Hyg. 2011;84: 200–207. 10.4269/ajtmh.2011.10-0503 21292885 PMC3029168

[2] PAHO. Integrated management strategy for arboviral disease prevention and control in the Americas. Washington, D.C., U.S.A: Pan American Health Organization, Pan American Sanitary Bureau, Regional Office of the World Health Organization; 2020.

[3] Pan American Health Organization. Plan continental de ampliación e intensificación del combate al Aedes aegypti. Informe de un grupo de trabajo, Caracas, Venezuela. Washington DC: PAHO; 1997. Report No.: OPS/HCP/HCT/90/97.

[4] Special Programme for Research and Training in Tropical Diseases, World Health Organization, editors. Dengue: guidelines for diagnosis, treatment, prevention, and control. New ed. Geneva: TDR: World Health Organization; 2009.

[5] WHO. Questions and answers on dengue vaccines: efficacy and longer-term safety of CYD-TDV. World Health Organization; 2015.

[6] WHO. Global Strategy for dengue prevention and control, 2012–2020. World Health Organization Report; 2012.

[7] Morrison AC, Zielinski-Gutierrez E, Scott TW, Rosenberg R. Defining Challenges and Proposing Solutions for Control of the Virus Vector Aedes aegypti. PLoS Med. 2008;5: e68. 10.1371/journal.pmed.0050068 18351798 PMC2267811

[8] Alonso PL, Tanner M. Public health challenges and prospects for malaria control and elimination. Nat Med. 2013;19: 150–155. 10.1038/nm.3077 23389615

[9] CDC. Surveillance and Control of Aedes aegypti and Aedes albopictus in the United States. Centers for Disease Control; 2015. Available: http://www.cdc.gov/chikungunya/pdfs/surveillance-and-control-of-aedes-aegypti-and-aedes-albopictus-us.pdf.

[10] K. Moise I, C. Zulu L, O. Fuller D, C. Beier J. Persistent Barriers to Implementing Efficacious Mosquito Control Activities in the Continental United States: Insights from Vector Control Experts. In: J. Rodriguez-Morales A, editor. Current Topics in Neglected Tropical Diseases. IntechOpen; 2019. 10.5772/intechopen.76774

[11] Eisen L, Beaty BJ, Morrison AC, Scott TW. Proactive Vector Control Strategies and Improved Monitoring and Evaluation Practices for Dengue Prevention. J Med Entomol. 2009;46: 1245–1255. 10.1603/033.046.0601 19960667

[12] Lippi CA, Mao L, Stewart-Ibarra AM, Heydari N, Ayala EB, Burkett-Cadena ND, et al. A network analysis framework to improve the delivery of mosquito abatement services in Machala, Ecuador. Int J Health Geogr. 2020;19. 10.1186/s12942-020-0196-6 32046732 PMC7014633

[13] Goodman H, Egizi A, Fonseca DM, Leisnham PT, LaDeau SL. Primary blood-hosts of mosquitoes are influenced by social and ecological conditions in a complex urban landscape. Parasit Vectors. 2018;11. 10.1186/s13071-018-2779-7 29631602 PMC5891940

[14] Stewart Ibarra AM, Ryan SJ, Beltrán E, Mejía R, Silva M, Muñoz Á. Dengue Vector Dynamics (Aedes aegypti) Influenced by Climate and Social Factors in Ecuador: Implications for Targeted Control. Mores CN, editor. PLoS ONE. 2013;8: e78263. 10.1371/journal.pone.0078263 24324542 PMC3855798

[15] Padmanabha H, Correa F, Rubio C, Baeza A, Osorio S, Mendez J, et al. Human Social Behavior and Demography Drive Patterns of Fine-Scale Dengue Transmission in Endemic Areas of Colombia. Paul R, editor. PLOS ONE. 2015;10: e0144451. 10.1371/journal.pone.0144451 26656072 PMC4684369

[16] Gubler DJ, Casta-Valez A. A program for prevention and control of epidemic dengue and dengue hemorrhagic fever in Puerto Rico and the U.S. Virgin Islands. Bull Pan Am Health Organ. 1991;25: 237–247. 1742570

[17] Focks Dana A. A review of entomological sampling methods and indicators for dengue vectors. Geneva: World Health Organization; 2004. Available: http://www.who.int/iris/handle/10665/68575. 10.1603/0022-2585-41.6.1123

[18] Cromwell EA, Stoddard ST, Barker CM, Van Rie A, Messer WB, Meshnick SR, et al. The relationship between entomological indicators of Aedes aegypti abundance and dengue virus infection. PLoS Negl Trop Dis. 2017;11: e0005429. 10.1371/journal.pntd.0005429 28333938 PMC5363802

[19] Takken W, editor. Ecological aspects for application of genetically modified mosquitoes. Dordrecht: Kluwer Acad. Publ; 2003.

[20] Lippi CA, Stewart-Ibarra AM, Muñoz ÁG, Borbor-Cordova MJ, Mejía R, Rivero K, et al. The Social and Spatial Ecology of Dengue Presence and Burden during an Outbreak in Guayaquil, Ecuador, 2012. Int J Environ Res Public Health. 2018;15. 10.3390/ijerph15040827 29690593 PMC5923869

[21] Lippi CA, Stewart-Ibarra AM, Loor MEFB, Zambrano JED, Lopez NAE, Blackburn JK, et al. Geographic shifts in Aedes aegypti habitat suitability in Ecuador using larval surveillance data and ecological niche modeling: Implications of climate change for public health vector control. PLoS Negl Trop Dis. 2019;13: e0007322. 10.1371/journal.pntd.0007322 30995228 PMC6488096

[22] Waldman EA, Díaz-Quijano FA. Factors Associated with Dengue Mortality in Latin America and the Caribbean, 1995–2009: An Ecological Study. Am J Trop Med Hyg. 2012;86: 328–334. 10.4269/ajtmh.2012.11-0074 22302870 PMC3269288

[23] Zellweger RM, Cano J, Mangeas M, Taglioni F, Mercier A, Despinoy M, et al. Socioeconomic and environmental determinants of dengue transmission in an urban setting: An ecological study in Nouméa, New Caledonia. Messer WB, editor. PLoS Negl Trop Dis. 2017;11: e0005471. 10.1371/journal.pntd.0005471 28369149 PMC5395238

[24] Dhewantara PW, Marina R, Puspita T, Ariati Y, Purwanto E, Hananto M, et al. Spatial and temporal variation of dengue incidence in the island of Bali, Indonesia: An ecological study. Travel Med Infect Dis. 2019;32: 101437. 10.1016/j.tmaid.2019.06.008 31362115

[25] Stewart Ibarra AM, Luzadis VA, Borbor Cordova MJ, Silva M, Ordoñez T, Beltrán Ayala E, et al. A social-ecological analysis of community perceptions of dengue fever and Aedes aegypti in Machala, Ecuador. BMC Public Health. 2014;14. 10.1186/1471-2458-14-1135 25370883 PMC4240812

[26] Ministerio de Salud Publica. Proyecto de Vigilancia y Control de Vectores para la Prevencion de al Transmision de Enfermedades Metaxenicas en el Ecuador. Servicio Nacional de Control de Enfermedades Transmitidas por Vectores Artropodos (SNEM), Ministerio de Salud Publica, Ecuador; 2013. Available: http://instituciones.msp.gob.ec/dps/snem/images/proyectocontroldevectoresmetaxenicas.pdf.

[27] Stewart-Ibarra AM, Ryan SJ, Kenneson A, King CA, Abbott M, Barbachano-Guerrero A, et al. The Burden of Dengue Fever and Chikungunya in Southern Coastal Ecuador: Epidemiology, Clinical Presentation, and Phylogenetics from the First Two Years of a Prospective Study. Am J Trop Med Hyg. 2018;98: 1444–1459. 10.4269/ajtmh.17-0762 29512482 PMC5953373

[28] Alava A., Mosquera C., Vargas W., Real J. Dengue en el Ecuador 1989–2002. Rev Ecuat Hig Med Trop. 2005;42: 11–34.

[29] INEC. Proyección de la Población Ecuatoriana, por años calendario, según cantones 2010–2020. Instituto Nacional de Estadística y Censos, Quito, Ecuador; 2019.

[30] Stewart-Ibarra AM, Muñoz ÁG, Ryan SJ, Ayala EB, Borbor-Cordova MJ, Finkelstein JL, et al. Spatiotemporal clustering, climate periodicity, and social-ecological risk factors for dengue during an outbreak in Machala, Ecuador, in 2010. BMC Infect Dis. 2014;14. 10.1186/s12879-014-0610-4 25420543 PMC4264610

[31] Quintero J, Brochero H, Manrique-Saide P, Barrera-Pérez M, Basso C, Romero S, et al. Ecological, biological and social dimensions of dengue vector breeding in five urban settings of Latin America: a multi-country study. BMC Infect Dis. 2014;14. 10.1186/1471-2334-14-38 24447796 PMC3904013

[32] Heydari N, Larsen D, Neira M, Beltrán Ayala E, Fernandez P, Adrian J, et al. Household Dengue Prevention Interventions, Expenditures, and Barriers to Aedes aegypti Control in Machala, Ecuador. Int J Environ Res Public Health. 2017;14: 196. 10.3390/ijerph14020196 28212349 PMC5334750

[33] Lowe R, Stewart-Ibarra AM, Petrova D, García-Díez M, Borbor-Cordova MJ, Mejía R, et al. Climate services for health: predicting the evolution of the 2016 dengue season in Machala, Ecuador. Lancet Planet Health. 2017;1: e142–e151. 10.1016/S2542-5196(17)30064-5 29851600

[34] Stewart-Ibarra AM, Lowe R. Climate and Non-Climate Drivers of Dengue Epidemics in Southern Coastal Ecuador. Am J Trop Med Hyg. 2013;88: 971–981. 10.4269/ajtmh.12-0478 23478584 PMC3752767

[35] The Modifiable Areal Unit Problem. Exploring Spatial Scale in Geography. Chichester, UK: John Wiley & Sons, Ltd; 2014. pp. 29–44. 10.1002/9781118526729.ch3

[36] Martínez-Bello DA, López-Quílez A, Torres Prieto A. Relative risk estimation of dengue disease at small spatial scale. Int J Health Geogr. 2017;16. 10.1186/s12942-017-0104-x 28810908 PMC5558735

[37] Khormi HM, Kumar L. The importance of appropriate temporal and spatial scales for dengue fever control and management. Sci Total Environ. 2012;430: 144–149. 10.1016/j.scitotenv.2012.05.001 22634561

[38] Turell MJ, Dohm DJ, Sardelis MR, O’Guinn ML, Andreadis TG, Blow JA. An update on the potential of North American mosquitoes (Diptera: Culicidae) to transmit West Nile virus. J Med Entomol. 2005;42. 10.1093/jmedent/42.1.57 15691009

[39] Kenneson A, Beltrán-Ayala E, Borbor-Cordova MJ, Polhemus ME, Ryan SJ, Endy TP, et al. Social-ecological factors and preventive actions decrease the risk of dengue infection at the household-level: Results from a prospective dengue surveillance study in Machala, Ecuador. Messer WB, editor. PLoS Negl Trop Dis. 2017;11: e0006150. 10.1371/journal.pntd.0006150 29253873 PMC5771672

[40] Kenneson A, Beltran Ayala EF, Borbor Cordova MJ, Polhemus ME, Ryan SJ, Endy TP, et al. S1 Text. Household survey instrument in English. PLOS Neglected Tropical Diseases; 2017. Available: 10.1371/journal.pntd.0006150.s002. PMC5771672 29253873

[41] Vazquez-Prokopec GM, Galvin WA, Kelly R, Kitron U. A new, cost-effective, battery-powered aspirator for adult mosquito collections. J Med Entomol. 2009;46: 1256–1259. 10.1603/033.046.0602 19960668 PMC2800949

[42] Hox JJ, Moerbeek M, van de Schoot R. Multilevel Analysis: Techniques and Applications. 3rd ed. Third edition. | New York, NY: Routledge, 2017. |: Routledge; 2017. 10.4324/9781315650982

[43] Chatterjee S, Hadi AS. Analysis of Collinear Data. Regression Analysis by Example. Hoboken, NJ, USA: John Wiley & Sons, Inc.; 2006. pp. 221–258. 10.1002/0470055464.ch9

[44] Ryan S, Lippi C, Nightingale R, Hamerlinck G, Borbor-Cordova M, Cruz B M, et al. Socio-Ecological Factors Associated with Dengue Risk and Aedes aegypti Presence in the Galápagos Islands, Ecuador. Int J Environ Res Public Health. 2019;16: 682. 10.3390/ijerph16050682 30813558 PMC6427784

[45] Getis A. A History of the Concept of Spatial Autocorrelation: A Geographer’s Perspective. Geogr Anal. 2008;40: 297–309. 10.1111/j.1538-4632.2008.00727.x

[46] Baly A, Toledo ME, Rodriguez K, Benitez JR, Rodriguez M, Boelaert M, et al. Costs of dengue prevention and incremental cost of dengue outbreak control in Guantanamo, Cuba: Costs of dengue in Guantanamo. Trop Med Int Health. 2012;17: 123–132. 10.1111/j.1365-3156.2011.02881.x 21906216

[47] Guzmán MG, Kourí G. Dengue diagnosis, advances and challenges. Int J Infect Dis. 2004;8: 69–80. 10.1016/j.ijid.2003.03.003 14732325

[48] Adin A, Martínez-Bello DA, López-Quílez A, Ugarte MD. Two-level resolution of relative risk of dengue disease in a hyperendemic city of Colombia. Schieffelin J, editor. PLOS ONE. 2018;13: e0203382. 10.1371/journal.pone.0203382 30204762 PMC6133285

[49] Fuentes-Vallejo M. Space and space-time distributions of dengue in a hyper-endemic urban space: the case of Girardot, Colombia. BMC Infect Dis. 2017;17. 10.1186/s12879-017-2610-7 28738782 PMC5525249

[50] Stoddard ST, Forshey BM, Morrison AC, Paz-Soldan VA, Vazquez-Prokopec GM, Astete H, et al. House-to-house human movement drives dengue virus transmission. Proc Natl Acad Sci. 2013;110: 994–999. 10.1073/pnas.1213349110 23277539 PMC3549073

[51] Reiner RC, Stoddard ST, Scott TW. Socially structured human movement shapes dengue transmission despite the diffusive effect of mosquito dispersal. Epidemics. 2014;6: 30–36. 10.1016/j.epidem.2013.12.003 24593919 PMC3971836

[52] Pepin KM, Leach CB, Marques-Toledo C, Laass KH, Paixao KS, Luis AD, et al. Utility of mosquito surveillance data for spatial prioritization of vector control against dengue viruses in three Brazilian cities. Parasit Vectors. 2015;8: 98. 10.1186/s13071-015-0659-y 25889533 PMC4335543

[53] Schwab SR, Stone CM, Fonseca DM, Fefferman NH. The importance of being urgent: The impact of surveillance target and scale on mosquito-borne disease control. Epidemics. 2017 [cited 20 Mar 2018]. 10.1016/j.epidem.2017.12.004 29279187

[54] Koyoc-Cardeña E, Medina-Barreiro A, Cohuo-Rodríguez A, Pavía-Ruz N, Lenhart A, Ayora-Talavera G, et al. Estimating absolute indoor density of Aedes aegypti using removal sampling. Parasit Vectors. 2019;12. 10.1186/s13071-019-3503-y 31113454 PMC6528352

[55] LaCon G, Morrison AC, Astete H, Stoddard ST, Paz-Soldan VA, Elder JP, et al. Shifting Patterns of Aedes aegypti Fine Scale Spatial Clustering in Iquitos, Peru. Diuk-Wasser MA, editor. PLoS Negl Trop Dis. 2014;8: e3038. 10.1371/journal.pntd.0003038 25102062 PMC4125221

[56] Maciel-de-Freitas R, Eiras ÁE, Lourenço-de-Oliveira R. Field evaluation of effectiveness of the BG-Sentinel, a new trap for capturing adult Aedes aegypti (Diptera: Culicidae). Mem Inst Oswaldo Cruz. 2006;101: 321–325. 10.1590/s0074-02762006000300017 16862330

[57] Williams CR, Long SA, Russell RC, Ritchie SA. Field efficacy of the BG-Sentinel compared with CDC backpack aspirators and CO_2_ -baited EVS traps for collection of adult Aedes aegypti in Cairns, Queensland, Australia. J Am Mosq Control Assoc. 2006;22: 296–300. 10.2987/8756-971X(2006)22[296:FEOTBC]2.0.CO;2 17019776

[58] Eisen L, García-Rejón JE, Gómez-Carro S, Nájera Vázquez M del R, Keefe TJ, Beaty BJ, et al. Temporal correlations between mosquito-based dengue virus surveillance measures or indoor mosquito abundance and dengue case numbers in Mérida City, México. J Med Entomol. 2014;51: 885–890. 10.1603/me14008 25118425 PMC4134096

[59] Eisenberg JNS, Schafrick NH, Milbrath MO, Berrocal VJ, Wilson ML. Spatial Clustering of Aedes aegypti Related to Breeding Container Characteristics in Coastal Ecuador: Implications for Dengue Control. Am J Trop Med Hyg. 2013;89: 758–765. 10.4269/ajtmh.12-0485 24002483 PMC3795109

[60] Reiter P, Lathrop S, Bunning M, Biggerstaff B, Singer D, Tiwari T, et al. Texas Lifestyle Limits Transmission of Dengue Virus. Emerg Infect Dis. 2003;9: 86–89. 10.3201/eid0901.020220 12533286 PMC2873752

[61] Waterman SH, Zielinski-Gutierrez E, Anaya-Lopez L, Brunkard JM, Smith B, Fournier M, et al. Epidemic Dengue and Dengue Hemorrhagic Fever at the Texas–Mexico Border: Results of a Household-based Seroepidemiologic Survey, December 2005. Am J Trop Med Hyg. 2008;78: 364–369. 10.4269/ajtmh.2008.78.364 18337327

[62] Demanou M, Pouillot R, Grandadam M, Boisier P, Kamgang B, Hervé JP, et al. Evidence of Dengue Virus Transmission and Factors Associated with the Presence of Anti-Dengue Virus Antibodies in Humans in Three Major Towns in Cameroon. Ribeiro GS, editor. PLoS Negl Trop Dis. 2014;8: e2950. 10.1371/journal.pntd.0002950 25009996 PMC4091864

[63] Krystosik A, Njoroge G, Odhiambo L, Forsyth JE, Mutuku F, LaBeaud AD. Solid Wastes Provide Breeding Sites, Burrows, and Food for Biological Disease Vectors, and Urban Zoonotic Reservoirs: A Call to Action for Solutions-Based Research. Front Public Health. 2020;7. 10.3389/fpubh.2019.00405 32010659 PMC6979070

[64] Walker N. The Hygienic House: Mosquito-Proofing with Screens. Am J Trop Med Hyg. 2010;83: 963–964. 10.4269/ajtmh.2010.10-0405 21036821 PMC2963953

[65] Ko Y-C, Chen M-J, Yeh S-M. The Predisposing and Protective Factors against Dengue Virus Transmission by Mosquito Vector. Am J Epidemiol. 1992;136: 214–220. 10.1093/oxfordjournals.aje.a116487 1415143

[66] Vazquez-Prokopec GM, Lenhart A, Manrique-Saide P. Housing improvement: a novel paradigm for urban vector-borne disease control? Trans R Soc Trop Med Hyg. 2016;110: 567–569. 10.1093/trstmh/trw070 27864518

[67] Bowman LR, Donegan S, McCall PJ. Is Dengue Vector Control Deficient in Effectiveness or Evidence?: Systematic Review and Meta-analysis. James AA, editor. PLoS Negl Trop Dis. 2016;10: e0004551. 10.1371/journal.pntd.0004551 26986468 PMC4795802

[68] PAHO. Re-emergence of dengue in the Americas. Epidemiol Bull Pan Am Health Organ. 1997;18: 95–98. 9376243

[69] Stewart-Ibarra A, Hargrave A, Diaz A, Kenneson A, Madden D, Romero M, et al. Psychological Distress and Zika, Dengue and Chikungunya Symptoms Following the 2016 Earthquake in Bahía de Caráquez, Ecuador. Int J Environ Res Public Health. 2017;14: 1516. 10.3390/ijerph14121516 29206195 PMC5750934

[70] Farinelli EC, Baquero OS, Stephan C, Chiaravalloti-Neto F. Low socioeconomic condition and the risk of dengue fever: A direct relationship. Acta Trop. 2018;180: 47–57. 10.1016/j.actatropica.2018.01.005 29352990

[71] NIOSH. Mosquito-borne diseases. National Institute for Occupational Safety and Health, Centers for Disease Control and Prevention; 2016.

[72] Potter A, Jardine A, Neville PJ. A Survey of Knowledge, Attitudes, and Practices in Relation to Mosquitoes and Mosquito-Borne Disease in Western Australia. Front Public Health. 2016;4. 10.3389/fpubh.2016.00032 26973827 PMC4770046

[73] Guttman N, Salmon CT. Guilt, Fear, Stigma and Knowledge Gaps: Ethical Issues in Public Health Communication Interventions. Bioethics. 2004;18: 531–552. 10.1111/j.1467-8519.2004.00415.x 15580723

