# Supplementary material for: Exploring the utility of social-ecological and entomological risk factors for dengue infection as surveillance indicators in the dengue hyper-endemic city of Machala, Ecuador
Source: PLoS Negl Trop Dis. 2021 Mar 19;15(3):e0009257. doi: 10.1371/journal.pntd.0009257 (PMC8011822; doi:10.1371/journal.pntd.0009257)
Supplement: S1 Table — (DOCX) [file pntd.0009257.s001.docx]

S1 Table. Description of social-ecological variables collected in Machala via household surveys.

| **Housing Conditions** | |
| --- | --- |
| **Variable** | **Description** |
| Good Condition Housing | Housing is newer construction and well maintained |
| Poor Condition Housing | Housing is old construction that has not been maintained, unpainted, visible damage to structure |
| Cane House Construction | Housing made of cane or bamboo |
| Wood House Construction | Housing made of wood material |
| House is Rented | House is rented, not purchased |
| Piped Water in Household | The household has access to running water |
| Interruptions in Piped Water | Household experiences daily or weekly interruptions in water service |
| Municipal Garbage Collection | Household has access to garbage collection services at least once a week |
| Municipal Sewage | Household is connected to city sewage |
| Septic Tank | Household is connected to a septic system |
| Air Conditioning | Air conditioning unit used to ventilate house |
| Uses a Fan | A fan is used to ventilate the house |
| Screens on Windows | Screens are present on all windows |
| Window Screen in Good Condition | If present, window screening is in good condition with no holes |
| Access to Paved Roads | Principal access to household is a paved road |
| Standing Water Present | Standing water was observed on the property |
| Adjacent to Abandoned Housing | Abandoned properties and households are adjacent to the household |
| Patio Present | The household has a patio area |
| Patio in Bad Condition | Patio is not maintained, with overgrown vegetation and garbage present |
| Patio Shaded (> 50%) | More than half of the patio area is covered by shade producing structures including greenhouse cloth and large tree canopies |
| **Household Demographics and Practices** | |
| Head of Household (HOH) Employed | Head of household is employed |
| HOH male | Head of household is male |
| HOH Earns Less than Minimum Wage | Head of household earns less than the local minimum wage |
| HOH Has Secondary Education | Head of household has completed secondary education |
| Stores Water | Members of the household store water |
| Know mosquitoes transmit dengue | Head of household knows that mosquitoes transmit dengue virus |
| Know standing water produces mosquitoes | Head of household knows that standing water produces mosquitoes |
| Uses Larvicide | Household uses larvicidal product (BTI) to control mosquitoes |
| Uses Abate | Household uses Abate/temefos to control mosquitoes |
